# The Cyclic AMP Receptor Protein, Crp, Is Required for the Decolorization of Acid Yellow 36 in *Shewanella putrefaciens* CN32

**DOI:** 10.3389/fmicb.2020.596372

**Published:** 2020-12-09

**Authors:** Weijie Liu, Ying Chen, Xuge Zhou, Jiawen Liu, Jingrong Zhu, Shiwei Wang, Cong Liu, Di Sun

**Affiliations:** ^1^Jiangsu Key Laboratory of Phylogenomics and Comparative Genomics, School of Life Sciences, Jiangsu Normal University, Xuzhou, China; ^2^Key Laboratory of Resources Biology and Biotechnology in Western China, Ministry of Education, College of Life Science, Northwest University, Xi’an, China

**Keywords:** *Shewanella putrefaciens* CN32, decolorization, azo dye, acid yellow, Crp

## Abstract

*Shewanella* shows good application potentials in the decolorization and detoxification of azo dye wastewater. However, the molecular mechanism of decolorization is still lacking. In this study, it was found that *Shewanella putrefaciens* CN32 exhibited good decolorization ability to various azo dyes, and a global regulatory protein cAMP receptor protein (Crp) was identified to be required for the decolorization of acid yellow 36 (AY) by constructing a transposon mutant library. Then, the molecular mechanism of AY decolorization regulated by Crp was further investigated. RT-qPCR and electrophoretic mobility shift assay (EMSA) results showed that Crp was able to directly bind to the promoter region of the *cymA* gene and promote its expression. Riboflavin acting as an electron shuttle could accelerate the AY decolorization efficiency of *S. putrefaciens* CN32 wild-type (WT) but did not show a promoting effect to Δ*crp* mutant and Δ*cymA* mutant, further confirming that Crp promotes the decolorization through regulating electron transport chains. Moreover, the mutant with *cymA* overexpression could slightly enhance the AY decolorization efficiency compared with the WT strain. In addition, it was found that MtrA, MtrB, and MtrC partially contribute to the electron transfer from CymA to dye molecules, and other main electron transport chains need to be identified in future experiments. This study revealed the molecular mechanism of a global regulator Crp regulating the decolorization of azo dye, which is helpful in understanding the relationship between the decolorization and other metabolic processes in *S. putrefaciens* CN32.

## Introduction

With the rapid development of the textile, printing, and dyeing industries, more than 700,000 tons of commercial synthetic dyes are produced worldwide each year (Guo et al., 2020b), of which azo dyes account for 60–70% (Hameed and Ismail, 2018; Kong et al., 2018), mainly due to their properties of low cost, easy synthesis, high coloring efficiency, and good stability to various oxidizing agents. The dyeing industries consume huge amounts of azo dyes, and about 10–15% of dye molecules that are not effectively bound to clothing is discharged into the surrounding environment in the form of dyeing wastewater (Zahir et al., 2014; Zhang et al., 2019), causing serious environmental pollution (Yesilada et al., 2003). Dyeing wastewater not only affects the transparency of the surrounding water but also poses a serious threat to the ecological environment and even human health because azo dye molecules and their degradation products have strong mutagenic, teratogenic, and carcinogenic effects (Guo et al., 2020a; Samir et al., 2020). The most remarkable characteristic of azo dyes is that their molecules contain one or more azo groups (Yan et al., 2012). Because of their stubborn structural properties, azo dyes are very stable in nature and difficult to degrade (Liu W. et al., 2017). Therefore, decolorization and detoxification are very necessary before the discharge of azo dye-containing wastewater (Liu et al., 2017b).

Physical, chemical, and biological strategies are generally applied to treat azo dye-containing wastewater (Pandey et al., 2007). Compared with physicochemical methods, which are limited by high energy consumption and secondary pollution, biological methods attracted more and more attention due to their advantages of high decolorization efficiency, low operation cost, and environmental friendliness (Saratale et al., 2011; Samir et al., 2020; Wang et al., 2020). The mechanism of biological decolorization of azo dyes mainly includes bioflocculation (Liu et al., 2009), biological adsorption (Saratale et al., 2011), electron reduction (Cai et al., 2012), and enzymatic degradation (Dawkar et al., 2010; Baweja et al., 2016; Yang et al., 2018, 2020). Moreover, the decolorization efficiency of azo dyes can also be enhanced by the combined use of different biological methods at the same time. For example, the degradation efficiency of insoluble Sudan red can be accelerated by the synergistic effect of enzyme-catalyzed biodegradation and non-specific reductive decolorization (Liu et al., 2018). In addition, much effort has been made to improve the decolorization efficiency of azo dyes (Imran et al., 2016). For example, some electron shuttles, such as riboflavin and methylene blue, have been found to be able to promote electron transfer from the cell surface to the dye molecules, thereby accelerating the biodegradation of azo dyes (Liu et al., 2016).

Many strains have been reported to be able to decolorize azo dyes; among them, *Shewanella* strains are the most concerned species due to their excellent decolorization performance (Cai et al., 2012; Liu et al., 2016, 2018). *Shewanella*, as a species of facultative anaerobic bacteria with remarkable respiratory pathways, is able to utilize various terminal electron acceptors under anaerobic conditions, including various pollutants, such as azo dyes and heavy metal ions (Fredrickson et al., 2008; Ding et al., 2014). Therefore, *Shewanella* strains show good potential in the field of environmental remediation. In *Shewanella*, many components in the electron transfer pathway are necessary for its decolorization ability (Cai et al., 2012; Xiao et al., 2012; Liu et al., 2016). In the cytomembrane, the tetraheme c-type cytochrome CymA receives electrons from the quinone pool and then transfers them to multiple respiratory pathways, such as Mtr, Dms, NarfA/B, and NrfA pathways (Schwalb et al., 2003; Gao et al., 2009). In *Shewanella oneidensis* MR-1, the Mtr respiratory pathway, including MtrA, MtrB, MtrC, and OmcA, has been found to be involved in decolorization processes of various azo dyes (Cai et al., 2012; Xiao et al., 2012). However, studies on the molecular mechanism of the decolorization of azo dyes by *Shewanella* are still lacking, especially on the global regulatory factors related to the decolorization of azo dyes.

In this study, cAMP receptor protein (Crp), a global transcription regulator (Gao et al., 2012), was found to be essential for the decolorization of azo dyes in *Shewanella putrefaciens* CN32 through the construction of a transposon mutant library and the selection of mutants with different decolorization abilities and transposon locations. And then, the molecular mechanism of the decolorization of azo dyes regulated by Crp was also investigated. We found that, with the assistance of cAMP, Crp was able to directly bind to the promoter region of the *cymA* gene and promote its expression, thereby promoting decolorization through regulating electron transport chains. Thus, this study has a certain theoretical significance for revealing the molecular mechanism of the decolorization of azo dyes by *Shewanella* strains.

## Materials and Methods

### Bacterial Strains, Plasmids, Primers, and Culture Conditions

The strains and plasmids used or constructed in this study are shown in Table 1, and the primers used in this study are listed in Table 2. *Escherichia coli* and *S. putrefaciens* CN32 strains were grown aerobically at 37°C and 30°C, respectively, in Luria–Bertani (LB) medium which contains tryptone 10 g/L, yeast extract 5 g/L, and NaCl 10 g/L, for genetic manipulation. If necessary, kanamycin of 50 μg/ml was added into the medium. The decolorization medium for *S. putrefaciens* CN32 consisted of yeast extract 2 g/L, NH_4_Cl 1 g/L, NaCl 0.5 g/L, Na_2_HPO_4_⋅12H_2_O 7.52 g/L, NaH_2_PO_4_⋅2H_2_O 7.13 g/L, and filter-sterilized sodium lactate 20 mM. The transformants were screened on the LKT medium, which is based on the LB medium added with 50 μg/ml of kanamycin and 20 μg/ml of potassium tellurite.

**TABLE 1:** The strains and plasmids used in this study.

Strains/plasmids	Descriptions	Sources
***E. coli* strains**		
DH5α	Host for cloning	Lab stock
BL21(DE3)	Expression host for pET-28a(+)	Lab stock
UQ3021	DH5a/λ*pir*	Larsen et al., 2002
UQ3022	UQ3021/pRL27, Km^r^	Larsen et al., 2002
***S. putrefaciens* strains**		
CN32	Wild type	Lab stock
Δ*crp*	In-frame deletion mutant of *crp* (*sputcn32_0652*) in CN32	This study
C-*crp*	Δ*crp* mutant carrying complement pBBR1MCS-2-P*_*aacC*1_*-*crp*	This study
Δ*cymA*	In-frame deletion mutant of *cymA* (*sputcn32_0286*) in CN32	This study
Δ*crp*Δ*cymA*	Mutant with in-frame deletion of *crp* and *cymA* in CN32	This study
C-*cymA*	Δ*cymA* mutant carrying complement pBBR1MCS-2-P*_*aacC*1_*-*cymA*	This study
O-*cymA*	CN32 with overexpression plasmid pBBR1MCS-2-P*_*aacC*1_*-*cymA*	This study
Δ*mtrA*	In-frame deletion mutant of *mtrA* (*sputcn32_1477*) in CN32	This study
Δ*mtrB*	In-frame deletion mutant of *mtrB* (*sputcn32_1476*) in CN32	This study
Δ*mtrC*	In-frame deletion mutant of *mtrC* (*sputcn32_1478*) in CN32	This study
Δ*mtrCAB*	Mutant with in-frame deletion of *mtrC*, *mtrA*, and *mtrB* in CN32	This study
Δ*undA*	In-frame deletion mutant of *undA* (*sputcn32_1479*) in CN32	This study
Δ*undA*Δmtr*CAB*	Mutant with in-frame deletion of *undA*, *mtrC*, *mtrA*, and *mtrB*	This study
**Plasmids**		
pRL27	Contains mini-Tn5 transposon (*ori*R6K) delivery vector, Km^r^	Larsen et al., 2002
pK19*mobsacB*	Suicide plasmid for strain CN32, Km^r^	Schäfer et al., 1994
pRK2013	Helper plasmid in triparental conjugation	Figurski and Helinski, 1979
pBBR1MCS-2-P*_*aacC*1_*	Broad-host-range plasmid with the promoter of *aacC1*; Km^r^	Liu et al., 2017a
pET-28a(+)	Vector for protein overexpression in BL21(DE3), Km^r^	Lab stock

**TABLE 2:** The primer sequences used in this study.

Primers	Sequences (5′–3′)	Functions or target genes
Tn5-seqF	CAGCAACACC TTCTTCACGA	For Tn5 Sequence
Tn5-seqR	AACAAGCCAGG GATGTAACG	For Tn5 Sequence
Crp-D5F	CTCAAAGAATTCTAAG ATGAGTCCAATCACTGTGCCC	*crp* deletion
Crp-D5R	GTATACTCTAGAGGTAC CCGTTAAGTTAGTCTTCAGC	*crp* deletion
Crp-D3F	TTTTACTCTAGAGATGTAA TAAAGGGTATCTGAATCT	*crp* deletion
Crp-D3R	AGGCAGAAGCTTCAGCGA GGTTATCTAAATTAGTGGG	*crp* deletion
Crp-UF	GTTGGATACACCAGTG CGAACAGAC	*crp* deletion
Crp-DR	TCTAAACTAAGACT TCTATCAAGTT	*crp* deletion
Crp-OF	CCAGCATGATATG TTCAAGATCTT	*crp* deletion
Crp-OR	GCAGCACTAAAAT CACCAATTTCT	*crp* deletion
Crp-InF	CCAATCTCTTGA CGAGTGATCTTG	*crp* deletion
Crp-InR	AAGGTTCTGTTG CCGTATTGATTAA	*crp* deletion
Crp-CF	CAAAGGGATCCCGA CCACACCATAAAGTTAGCCTG	*crp* complementation
Crp-CR	TTTAATGAATTCGAA ACAGGCTTAAATCAAGCTGAAG	*crp* complementation
Crp-EF	ATCAACGGATCCATGGC TCTGATTGGTAAGCCAAAACC	express His_6_-Crp protein
Crp-ER	TGGCCTAAGCTTAGAAT TTATGCTAGGCCACTTTAATG	express His_6_-Crp protein
CymA-D5F	CCGGAATTCCCATTG CAGTATCGCTTATG	*cymA* deletion
CymA-D5R	GATACAGAACT GATCCGTACTTG	*cymA* deletion
CymA-D3F	GCTCACCCATA TCCAAAAG	*cymA* deletion
CymA-D3R	AAAACTGCAGC TACCTATCCAAGATCTCGAAG	*cymA* deletion
CymA-UF	TATTGTCCTGAT AGTTAGAGCT	*cymA* deletion
CymA-DR	CCTGTTAGTTTA TCGTCAGC	*cymA* deletion
CymA-OF	GCCGAAGACAAA GAGATAG	*cymA* deletion
CymA-OR	AAACCGCCAAAA ATAAAC	*cymA* deletion
CymA-InF	CCTGTCACAGC AACCATT	*cymA* deletion
CymA-InR	CTGCGGAAATA CTTAAGTGC	*cymA* deletion
CymA-CF	TTCTAAGGATCCTAAGTGAAAT AGCATAAACTAGACTT	*cymA* complementation
CymA-CR	GTTCAAGAATTCGAATGAATCG CTAAAACCTATTATCC	*cymA* complementation
CymA-PF	GACTAAGAGTTTG ATGCATAAGTATT	*cymA* EMSA probe
CymA-PR	AATCATCAAACA ATCGCAAGTTAT	*cymA* EMSA probe
CymA-QF	GAACTGGCGT GCACTATT	*cymA* RT-qPCR
CymA-QR	ATACAGAACT GATCCGTACTTG	*cymA* RT-qPCR
16S QF	GCAGGCGGTTT GTTAAGCGAGATG	internal standard for RT-qPCR
16S QR	CTTCGCCACCGG TATTCCTCCAGA	internal standard for RT-qPCR
CymA-FootF	TGTAAAACGACGGCCA GTAATAATGAACGGCTCGAT	*cymA* DNase I footprinting
CymA-FootR	CAGGAAACAGCTATGACCAAT AGTGCACGCCAGTTC	*cymA* DNase I footprinting
MtrC-D5F	AAAACTGCAGCTGTGTTAG CTGTCATAATGA	*mtrC* deletion
MtrC-D5R	AAACTTGAAAT ATTGAAGCC	*mtrC* deletion
MtrC-D3F	GCAGTGCAG TCAGAAACC	*mtrC* deletion
MtrC-D3R	CCGGAATTCT GCTCACCTCCATGACAT	*mtrC* deletion
MtrC-UF	GCATTAACTTA AGTCGCCTC	*mtrC* deletion
MtrC-DR	TGTTCTTCATA ATAGGCTTCC	*mtrC* deletion
MtrC-OF	GTGGTTGTAGCA GTTGTCATAC	*mtrC* deletion
MtrC-OR	ATAATGCCCCTT ACTACTGG	*mtrC* deletion
MtrC-InF	GGTACCTACAG CTATGACTTCG	*mtrC* deletion
MtrC-InR	TGGTGTAATGT TTGGCGT	*mtrC* deletion
MtrA-D5F	CCGGAATTCAA GTATTGTTGACGGTAAGCT	*mtrA* deletion
MtrA-D5R	ATAACTCCCTT CAGCGAAC	*mtrA* deletion
MtrA-D3F	AATTGCCATA GTCAGGTTCA	*mtrA* deletion
MtrA-D3R	AAAACTGCAG CCAGATATCACATTGGTATTGTC	*mtrA* deletion
MtrA-UF	GCAGTGCAGTC AGAAACCT	*mtrA* deletion
MtrA-DR	AGCCCTTACAG CTCCATG	*mtrA* deletion
MtrA-OF	TTGAAATTATT ACTAACGTTGGCC	*mtrA* deletion
MtrA-OR	CTTGGCTCATT TGTCCCG	*mtrA* deletion
MtrA-InF	GCAATGAACC GATGATCAC	*mtrA* deletion
MtrA-InR	CGTGACAGGC ATAACAGGT	*mtrA* deletion
MtrB-D5F	CCGGAATTCG CCACCTTAGATAAAAAGTTCG	*mtrB* deletion
MtrB-D5R	AACGATGCA GCCCTTACAG	*mtrB* deletion
MtrB-D3F	GACGCCGCGA ATGATATC	*mtrB* deletion
MtrB-D3R	AAAACTGCAG TGCTAATAAAGATGTCATGGATGC	*mtrB* deletion
MtrB-UF	GCTGCTTAAAT TGCCATAGT	*mtrB* deletion
MtrB-DR	CTTGTCGTAGC GCTTAAAC	*mtrB* deletion
MtrB-OF	TTGCTACGAGT GCTCATG	*mtrB* deletion
MtrB-OR	GGCGACTTGCT TGTAATAT	*mtrB* deletion
MtrB-InF	GGCAAATTT GACGCTGAC	*mtrB* deletion
MtrB-InR	TACTATCCA GTTATCAGGCAATGT	*mtrB* deletion
UndA-D5F	AAAACTGCAGA CTGCGCCTATTGTAGCT	*undA* deletion
UndA-D5R	GGTCAGTACTA TATCAACGCTG	*undA* deletion
UndA-D3F	AACGGTGGTGT GTACAATG	*undA* deletion
UndA-D3R	TTGGATCCGCG AACATAGTTATTCAGTACAAT	*undA* deletion
UndA-UF	TGATGATGATTA CAACTATTGC	*undA* deletion
UndA-DR	TCTGTCAACTAT TGCTGCTT	*undA* deletion
UndA-OF	CCGATTTCAGA AATAATGC	*undA* deletion
UndA-OR	AGTAAAGACA GGTAGCGTGG	*undA* deletion
UndA-InF	TGGATCAGC TATATCAACTCAGT	*undA* deletion
UndA-InR	GGTGTATCAA GGTCGGGT	*undA* deletion
MtrC-D5F1	CCCAAGCTTC TGTGTTAGCTGTCATAATGA	*mtrCAB* deletion
MtrC-D5R1	TTGGATCCAAACT TGAAATATTGAAGCC	*mtrCAB* deletion
UndA-D5F1	CCCAAGCTTACTGCGCC TATTGTAGCT	*mtrCAB/undA* deletion
UndA-D5R1	TTGGATCCGGTCAGTA CTATATCAACGCTG	*mtrCAB/undA* deletion
MtrB-D3F1	TTGGATCCGACGCCGC GAATGATATC	*mtrCAB/undA* deletion
MtrB-D3R1	AAAACTGCAGTGCTAA TAAAGATGTCATGGATGC	*mtrCAB/undA* deletion

### Decolorization Assay of Azo Dyes

*S*. *putrefaciens* CN32 was cultured in LB medium for 12 h to obtain the seed culture and then inoculated into 60 ml serum vials containing 50 ml of the decolorization medium with azo dyes to an initial OD_600_ of 0.1. To ensure anaerobic condition, decolorization systems were purged with nitrogen gas for 2 min. Subsequently, serum vials were sealed with rubber stoppers immediately and then cultured in an incubator at 30°C without shaking for decolorization. The decolorization samples of different times were centrifuged at 10,000 rpm, 25°C for 1 min. The absorbance of the supernatant was determined at 497 nm for Congo red, 520 nm for amaranth, 414 nm for acid yellow 36 (AY), 465 nm for methyl orange, 618 nm for amino black, and 714 nm for naphthol green to determine the concentration of residual azo dyes. The decolorization rate (%) was calculated based on the following equation: (ODx − ODy)/ODx × 100%, where ODx and ODy refer to the absorbance of the initial and decolorized samples, respectively. Experiments were repeated independently at least three times.

### Transposon Mutagenesis and Location of Transposon Insertion Sites

*S. putrefaciens* CN32 (recipient strain) was mixed with *E. coli* UQ3022 (donor strain), which carries a plasmid pRL27 containing a mini-Tn5 transposon and a kanamycin resistance gene (Larsen et al., 2002). Both recipient strain and donor strain were cultured overnight in LB medium. The donor strain was washed with LB medium twice and mixed with the recipient strain in a 1:1 ratio. The mixture of these two strains was spotted on a solid LB agar plate and incubated at 30°C for 8 h. Then the cells were scraped off from the agar plate and plated onto an LB agar plate with 50 μg/ml of kanamycin and 20 μg/ml of potassium tellurite (to inhibit the cell growth of the donor strain) and incubated at 30°C for 36 h. Single black colonies presented were purified, and their decolorization abilities were determined in the decolorization medium with 200 mg/L AY. The mutants with significantly altered decolorization capacity were selected and preserved. Then, the genomic DNA of these mutants was extracted for localization of the transposon insertion site. The extracted genomic DNA was self-linked after digestion by *Bam*HI or *Spe*I and then transformed into *E. coli* UQ3021 (Larsen et al., 2002) and selected on the LB agar plate added with 50 μg/ml of kanamycin. Plasmids extracted from the transformant were sequenced using primer Tn5-seqF and Tn5-seqR and BLAST in NCBI to map the location of the mini-Tn5 transposon. The target genes inserted by the mini-Tn5 transposon were deleted to confirm its function in regulating AY decolorization in *S. putrefaciens* CN32.

### Construction of Deletion Mutants and Complementation Strains

**FIGURE 1:**
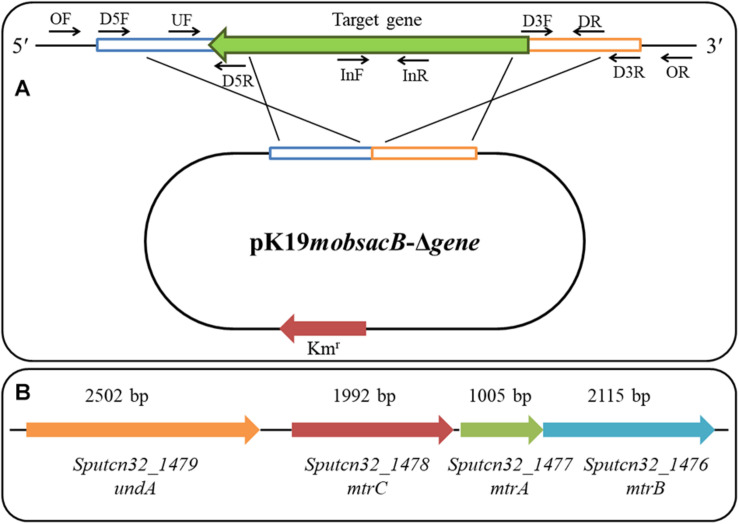
The primer positions for target gene in-frame deletion **(A)** and genomic arrangement of *mtrA, mtrB, mtrC*, and *undA* in *S. putrefaciens* CN32 **(B)**.

The in-frame deletion of mutants for *crp*, *mtrA*, *mtrB*, *mtrC*, *undA*, and *cymA* was performed based on the principle of homologous recombination. The primer positions for the target gene in-frame deletion are shown in Figure 1A, and the genomic arrangement of *mtrA*, *mtrB*, *mtrC*, *undA*, and *cymA* in *S. putrefaciens* CN32 is shown in Figure 1B. To delete the target gene, approximately 1,000-bp fragments upstream and downstream the targeted gene were amplified with respective primers D5F/D5R and D3F/D3R, using the *S. putrefaciens* CN32 genome DNA as a PCR template. These two fragments were ligated into the suicide vector pK19*mobsacB* after being digested by the corresponding restriction endonuclease (Schäfer et al., 1994). Then, the constructed plasmid was transformed into *E. coli* DH5α and introduced from *E. coli* DH5α into *S. putrefaciens* CN32 wild type (WT) using a helper plasmid, pRK2013, by triparental conjugation (Figurski and Helinski, 1979). The conjugation experiment was performed according to a previous study (Liu et al., 2017a). Briefly, the donor and recipient strains were conjugated in a 1:1 ratio and spotted on an LB agar plate. LKT medium plates were used to screen for transconjugants after conjugation for 8 h at 30°C. The single-crossover recombinant strain was selected using the primer UF/DR. After overnight culture in the LB medium added with 50 μg/ml of kanamycin, a single-crossover recombinant strain was transferred to a NaCl-free LB medium in a ratio of 0.1%, and the double-crossover disruptants were screened according to the sucrose sensitivity and finally checked by four pairs of primers: OF/OR, UF/OR, OF/DR, and InF/InR. All the mutants were confirmed by sequencing analysis. For the construction of the Δ*mtrCAB* mutant, upstream and downstream fragments were amplified with primers MtrC-D5F1/MtrC-D5R1 and MtrB-D3F1/MtrB-D3R1, respectively, and for the construction of Δ*undA*Δ*mtrCAB*, upstream and downstream fragments were amplified with primers UndA1-D5F1/UndA1-D5R1 and MtrB-D3F1/MtrB-D3R1, respectively.

To construct a complementation plasmid, a DNA fragment carrying the ribosome binding site (RBS) and open reading frame (ORF) of the targeted gene was amplified by primers CF/CR using the *S. putrefaciens* CN32 genome as a PCR template and then ligated with the plasmid pBBR1MCS-2-P*_*aacC*1_* with the corresponding restriction site (Liu et al., 2017a). And then the complementation plasmid was introduced into corresponding mutants for complementation or into *S. putrefaciens* CN32 WT for overexpression.

### RNA Extraction and Real-Time RT-PCR Analysis

In order to analyze the regulatory effect of Crp on the *cymA* gene, the seed samples of WT and Δ*crp* mutant were inoculated into the decolorization medium with 200 mg/L AY and cultured for 4 h. The cells were collected by centrifugation at 10,000 rpm, 4°C for 5 min, and RNA was extracted by the TRIzol reagent (Tiangen, China) according to the protocol provided by the manufacturer. Real-time RT-PCR was performed using primers CymA-QF/CymA-QR, with SYBR Green Master Mix (Biosharp, China) according to the specification from the manufacturer. Signal intensities of PCR products were standardized to those of the *16S rRNA* gene amplified with primers 16S QF/16S QR. The experiments were performed with at least three replicates.

### Purification of His_6_-Crp Protein

To prepare the His_6_-Crp protein, the coding region of *crp* was amplified by PCR using primers Crp-EF/Crp-ER. The PCR product was digested with *Bam*HI/*Hin*dIII and cloned into pET-28a (+) to generate pET28-*crp*. The resulting plasmid was confirmed by sequencing and then transformed into *E. coli* BL21(DE3) for overexpression of His_6_-Crp (six-histidine tag on the N-terminus of the Crp protein). After induction with 0.4 mM isopropyl β-D-thiogalactoside (IPTG) at 16°C for 20 h, the soluble recombinant His_6_-Crp protein was purified by Ni-agarose resin chromatography (CoWin Biosciences, China).

### Electrophoretic Mobility Shift Assay (EMSA)

DNA probe-carrying promoter regions of *cymA* were PCR-amplified by using primers CymA-PF and CymA-PR. The PCR products purified from agarose gel were labeled with digoxigenin (DIG) using terminal transferase. The 3′-terminal DIG-labeled probe of 0.15 nM was incubated with various quantities of His_6_-Crp and 1 μM cAMP in a binding reaction. EMSA experiments were operated as described previously (Sun et al., 2016). To confirm specificity of protein–DNA interaction, a 300-fold excess of unlabeled specific probe or non-specific DNA was added into the binding mixture before incubation.

### DNase I Footprinting

DNase I footprinting assay was carried out to determine the binding site of Crp in the promoter region of *cymA*. PCR was conducted using 5′-terminal FAM-labeled forward primer CymA-FootF and regular reverse primer CymA-FootR, and the PCR products were purified to obtain FAM-labeled footprinting probes. The mixtures of 20-μl volume containing various concentrations of purified His_6_-Crp, 300-ng probes, and 2 μM of cAMP were incubated at 25°C for 40 min to achieve the binding reaction of the Crp protein with the probe. Then 1 U of DNase I (NEB, United States) was added to the mixture and incubated at 37°C for 10 s. The DNase I treatment was terminated by adding 10 μl of 0.5 M EDTA solution and heated at 80°C for 10 min. The DNA fragments were purified and capillary sequenced in a 3730XL DNA Genetic Analyzer (ABI, United States). The data were processed and analyzed with the GeneMarker program, v2.2.0.

### Effect of Exogenous Riboflavin on Decolorization

In order to analyze the effect of exogenous riboflavin on AY decolorization of *S. putrefaciens* CN32 WT, Δ*crp*, and Δ*cymA*, the strains were cultured in LB medium to an OD_600_ of around 1.0 and used as seed culture. Then, the cells in seed culture were collected by centrifugation at 25°C, 8,000 rpm for 5 min, and were washed once using the decolorization base medium (decolorization medium without adding yeast extract). The washed cells were inoculated into 60 ml serum vials containing 50 ml of the decolorization base medium with 200 mg/L of AY to an initial OD_600_ of 0.1. After purging with nitrogen gas for 2 min, the decolorization ability of WT, Δ*crp*, and Δ*cymA* with and without adding 2 μM riboflavin was determined at different time intervals.

## Results and Discussion

### Decolorization of Various Azo Dyes by *S. putrefaciens* CN32

**FIGURE 2:**
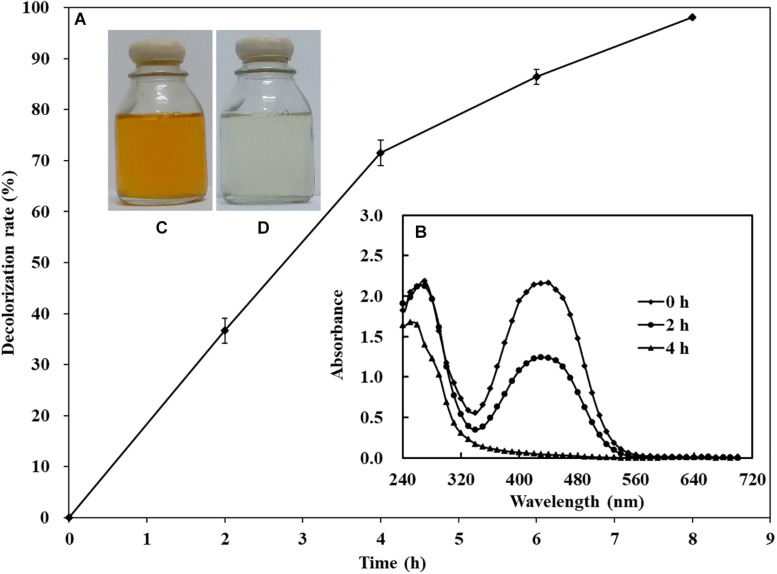
Decolorization of 200 mg/L of AY by *S. putrefaciens* CN32 **(A)**. Scanning spectrum analysis of 50 mg/L of AY decolorized by *S. putrefaciens* CN32 at different time intervals **(B)**. The image of 50 mg/L AY before **(C)** and after **(D)** decolorization.

The decolorization ability of *S. putrefaciens* CN32 to various azo dyes was analyzed under anaerobic condition (Table 3). The results showed that *S. putrefaciens* CN32 was able to decolorize all the tested azo dyes within 48 h. Especially to AY (Figure 2) and methyl orange, more than 90% of decolorization rates were achieved within 8 h. As shown in Figure 2, the scanning spectrum of AY and its degradation products in a range from 240 to 720 nm was investigated. It can be seen that the characteristic absorption peak at around 414 nm disappeared after decolorization of 4 h, suggesting that *S. putrefaciens* CN32 shows good potential in the treatment of azo dye-containing wastewater. In previous studies, several *Shewanella* strains have been used to decolorize azo dyes, such as *S. oneidensis* MR-1 (Liu et al., 2016; Li et al., 2018), *Shewanella decolorationis* S12 (Xu et al., 2007), *Shewanella* sp. RQs-106 (Zhou et al., 2018), *Shewanella aquimarina* (Meng et al., 2012), and *Shewanella algae* (Meng et al., 2014). In *S. oneidensis* MR-1, a transmembrane electron transport chain Mtr respiratory pathway, which includes cytochromes MtrC and OmcA and related proteins MtrA and MtrB, has been proven to be responsible for the anaerobic decolorization of azo dyes (Xiao et al., 2012). However, the further regulatory mechanism of the decolorization of azo dyes in *Shewanella* species is largely unknown. In this study, AY was selected for investigating the molecular mechanism of azo dye decolorization by *S. putrefaciens* CN32 due to its very high decolorization efficiency.

**TABLE 3:** Decolorization ability of *S. putrefaciens* CN32 to tested azo dyes.

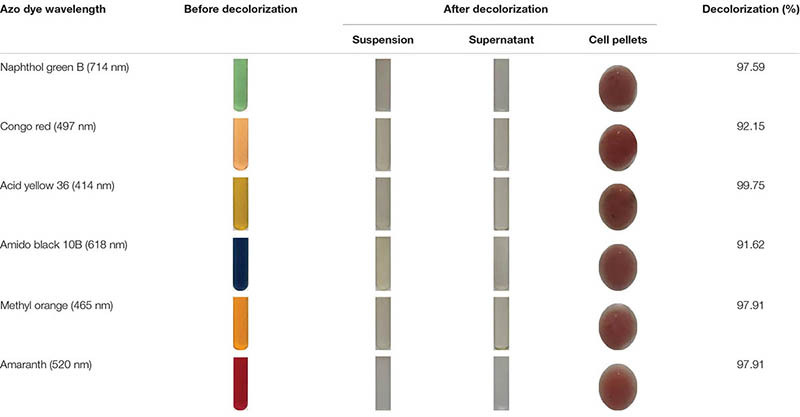

**The initial concentration of azo dyes is 50 mg/L. The images of azo dye solution obtained before decolorization and after 24 h of decolorization. The cell pellets were collected from decolorization solution using a 2 ml tube with 10,000 rpm.**

### Crp Promotes the Decolorization Ability of *S. putrefaciens* CN32 Under Anaerobic Conditions

**FIGURE 3:**
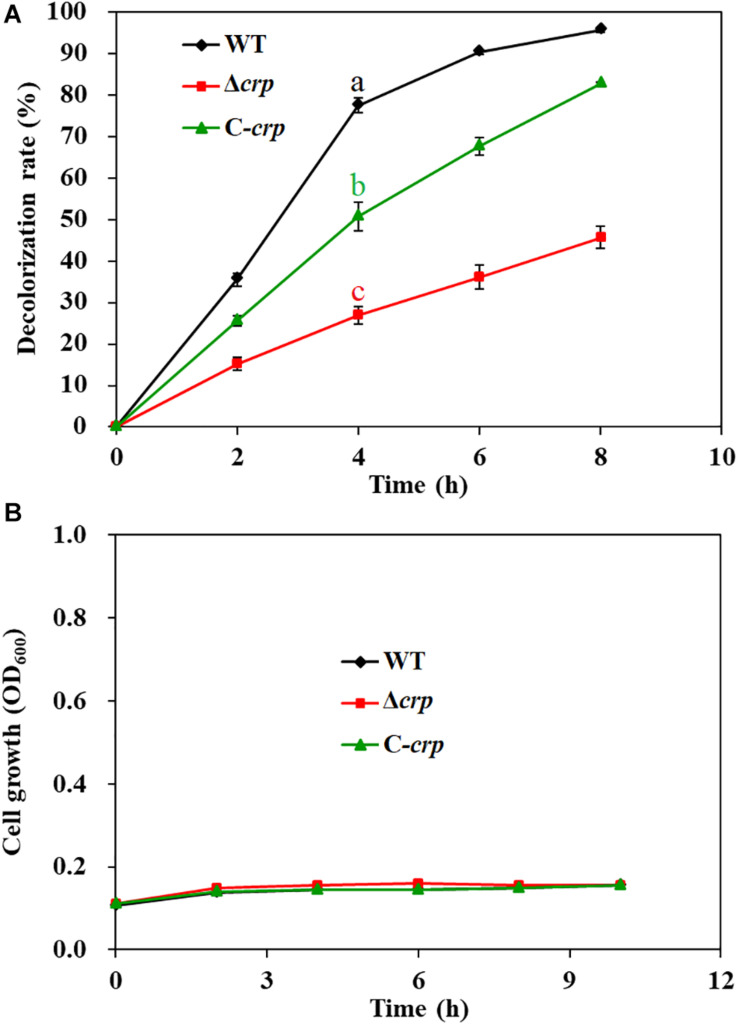
Decolorization of AY **(A)** and cell growth **(B)** of the WT, Δ*crp* mutant, and C-*crp* mutant. The initial concentration of AY is 200 mg/L. The cell growth was determined in the decolorization medium under anaerobic conditions. Values for decolorization and cell growth are means ± SD (*n* = 3). Significance analysis of decolorization rates of different strains at 4 h was performed using one-way analysis of variance (ANOVA) with the Tukey–Kramer comparison test (*p* < 0.05).

To identify the underlying decolorization mechanisms of AY by *S. putrefaciens* CN32 in anaerobic respiration, approximately 1,000 mini-Tn5 transposon-inserted mutants screened by a kanamycin agar plate were selected for decolorization ability analysis using 60 ml serum vials. Compared with the CN32 WT strain, the decolorization efficiencies of 10 transposon-inserted mutants were significantly altered (more than 50% change), in which three mutants that exhibited poor decolorization ability to AY were inserted into different sites of the *crp* gene (*sputcn32_0652*), suggesting that Crp may be involved in biodecolorization. Thus, an in-frame deletion mutant of the *crp* gene (Δ*crp*) and a complementation strain (C-*crp*) were constructed. As shown in Figure 3A, the anaerobic decolorization efficiency of the Δ*crp* mutant to AY was seriously decreased compared with the WT, and the C-*crp* mutant was obviously restored to the WT level. At the same time, the growth of WT, Δ*crp* mutant, and C-*crp* mutant was determined under the anaerobic decolorization condition. The results (Figure 3B) showed that the WT, Δ*crp* mutant, and C-*crp* mutant did not show obvious cell growth during the anaerobic AY decolorization process. Therefore, these results indicated that Crp promotes AY decolorization in *S. putrefaciens* CN32. Crp is a global regulatory factor which regulates different metabolic processes in many bacteria by forming complexes with cAMP; for example, Crp is involved in carbon catabolite repression in many bacteria (Deutscher et al., 2006); in *E. coli*, Crp regulates many stress responses to protect cells from harmful environments including starvation and osmotic shock (Battesti et al., 2011; Kalia et al., 2013); in *Pseudomonas aeruginosa*, Crp (Vfr) regulates biofilm formation through controlling type IV pili (Beatson et al., 2002; Persat et al., 2015). In this study, it was found that Crp is necessary for biological decolorization in *S. putrefaciens* CN32.

### CymA Is Necessary for the Anaerobic Decolorization of AY in *S. putrefaciens* CN32

**FIGURE 4:**
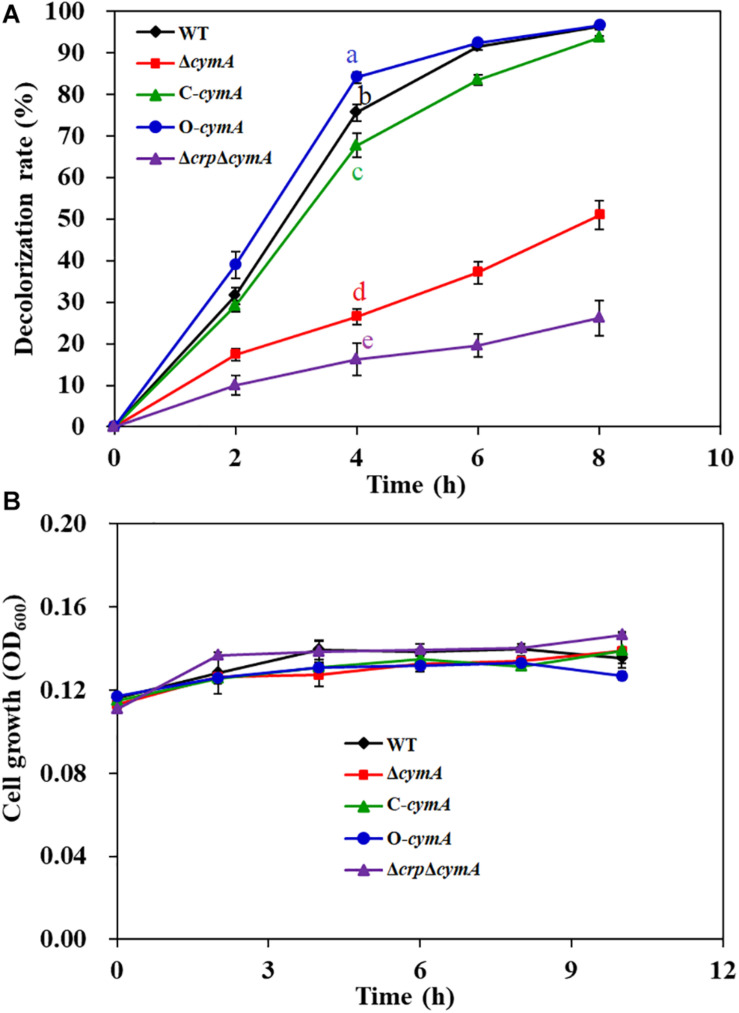
Decolorization of 200 mg/L of AY **(A)** and cell growth **(B)** of the WT, Δ*cymA* mutant, C-*cymA* mutant, and Δ*crp*Δ*cymA* mutant. Values for decolorization and cell growth are means ± SD (*n* = 3). The cell growth was determined in the decolorization medium under anaerobic conditions. The decolorization rates of different strains at 4 h were analyzed by ANOVA with the Tukey–Kramer comparison test (*p* < 0.05).

Except for the *crp* gene, two transposon-inserted mutants with a significant decrease in decolorization efficiency were identified with different insertion sites in the *cymA* gene (*sputcn32_0286*). CymA, a c-type cytochrome, is a component of the Mtr respiratory pathway, which is a critical transmembrane electron transfer channel in dissimilatory metal-reducing strains (Xiao et al., 2012). A previous study showed that the Δ*cymA* mutant of *S. oneidensis* MR-1 almost lost complete decolorization capability (Xiao et al., 2012). Genome analysis found that the amino acid sequence of CymA in *S. putrefaciens* CN32 exhibits 95.7% similarity to that in *S. oneidensis* MR-1. To confirm the function of CymA in *S. putrefaciens* CN32 during AY decolorization, an in-frame gene deletion mutant Δ*cymA* and a complementation strain C-*cymA* were constructed. The anaerobic decolorization ability of the Δ*cymA* mutant was seriously decreased compared with that of the WT strain (Figure 4A); the C-*cymA* mutant was obviously restored to the WT level. And the WT, Δ*cymA* mutant, and C-*cymA* mutant exhibited similar cell growth (Figure 4B), suggesting that CymA is necessary for the anaerobic decolorization of AY in *S. putrefaciens* CN32. As the Δ*cymA* mutant showed a similar phenotype to the Δ*crp* mutant, a double mutant Δ*crp*Δ*cymA* was constructed to analyze their relationship. The results showed that the Δ*crp*Δ*cymA* mutant exhibited a similar decolorization efficiency to the Δ*crp* and Δ*cymA* mutants, which is obviously lower than that of the WT strain, indicating that Crp and CymA may be involved in the same decolorization pathway.

### Crp Directly Activates the Transcription of *cymA*

**FIGURE 5:**
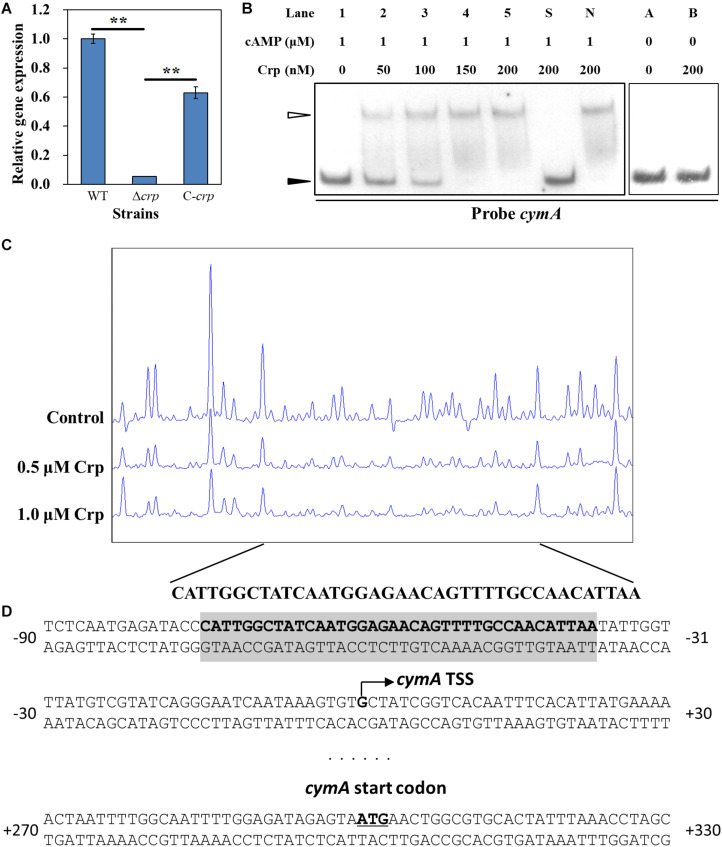
Regulatory effect of Crp on *cymA* gene. **(A)** The relative expression level of the *cymA* gene in the WT, Δ*crp* mutant, and C-*crp* mutant. *cymA* expression level in the WT strain was normalized as 1. Error bars indicate standard deviation of three samples. Significance analysis was performed using Student’s *t*-test; **p < 0.01. **(B)** EMSAs of the interaction of different concentrations of His_6_-Crp with DNA probes; 0.15 nM labeled probe and 1.0 μM cAMP were added to reaction mixtures with different concentrations of His_6_-Crp (Lanes 1, 2, 3, 4, and 5). A 300-fold-excess unlabeled specific competitor (Probe *cymA*, Lane S) and non-specific competitor (Lane N) were added to the mixture to perform specific or non-specific competition assays, respectively. Lanes A and B: Reaction mixtures without addition of cAMP used as negative controls. Filled triangles: Free probes. Empty triangle: Protein–DNA complex. **(C)** DNase I footprinting assay of different concentrations (0.5 and 1.0 μM) of His_6_-Crp and *cymA* in the upstream region. Each reaction mixture contains 300-ng FAM-labeled DNA probe and 2 μM cAMP. Control: No His_6_-Crp protein in the reaction mixture. The nucleotide sequences of the protected region were shown below the graph. **(D)** Nucleotide sequences of *cymA* promoter region and His_6_-Crp binding site. Bent arrow: Predicted *cymA* TSS. Shaded region: Region protected by Crp in the DNase I footprinting assay. Underline: *cymA* start codon. Numbers indicate the distance (nt) from TSS.

Crp is a critical global transcriptional regulator in prokaryotes through forming a complex with cAMP. In *S. oneidensis* MR-1, the complex of Crp and cAMP can regulate the transcription of multiple cytochrome c genes including *omcA* and *mtrC* (Kasai et al., 2015). However, the relationship between Crp and the *cymA* gene is still unclear. To determine whether Crp regulates the transcription of the *cymA* gene in *S. putrefaciens* CN32, real-time reverse-transcription PCR (RT-qPCR) was carried out (Figure 5A). Compared with that in the WT strain, the *cymA* expression level in the Δ*crp* mutant was significantly decreased, and almost no *cymA* expression was detected in the Δ*crp* mutant, indicating that Crp plays a critical role in activating the expression of the *cymA* gene. To identify the regulation that Crp exerts on the transcription of the *cymA* gene, EMSA was performed to investigate whether Crp directly binds to the upstream regions of the *cymA* gene. When a labeled DNA probe containing the region from −183 to −477 bp upstream of *cymA* was incubated with the Crp–cAMP complex, shifted bands were observed (Figure 5B). When there is no cAMP in the mixture, no shifted band was observed (Figure 5B). These findings suggested that the Crp–cAMP complex can bind to the promoter region of the *cymA* gene. Taken together, Crp directly activates the transcription of the *cymA* gene through forming a complex with cAMP. To further investigate the mechanism by which the Crp–cAMP complex activates *cymA*, a DNase I footprinting assay was carried out to identify the precise binding site of the Crp–cAMP complex at the upstream regions of the *cymA* gene, and a protected region from −375 to −338 nt upstream of the *cymA* start codon was revealed (Figure 5C). Subsequently, the transcription start site (TSS) of *cymA* was predicted using an online BDGM promoter prediction tool^1^. A possible TSS was found to be a “G” located at 300 nt upstream of the *cymA* start codon (Figure 5D). Based on the above results, it is possible that the Crp–cAMP complex activates *cymA* expression by recruiting the RNA polymerase to the promoter region of *cymA*.

### Effect of Exogenous Riboflavin on Decolorization

**FIGURE 6:**
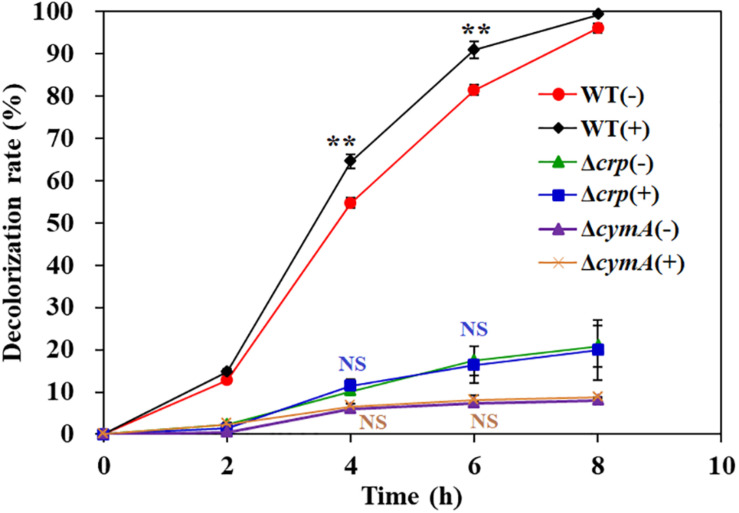
The effect of exogenous riboflavin on AY decolorization. Values are means ± SD (*n* = 3). The cells were cultured in decolorization medium with (+) or without (–) adding 2 μM riboflavin. The initial AY concentration of is 200 mg/L. Significance analysis of decolorization rates of the same strain with and without riboflavin was performed using Student’s *t*-test; asterisks **p < 0.01, and NS indicates not significant.

Previous studies have reported that flavins produced from *Shewanella* genus (Marsili et al., 2008) and other chemical substances, such as methylene blue (Liu et al., 2016) and humic acids (Liu et al., 2011), were able to act as shuttles to accelerate the electron transfer from the cell surface to pollutant molecules, such as azo dyes and heavy metal ions. In order to analyze the effect of electron shuttle on AY decolorization by *S. putrefaciens* CN32, 2 μM riboflavin was added into the decolorization systems of WT, Δ*crp*, and Δ*cymA*. The results showed that riboflavin could significantly improve the decolorization efficiency of the WT strain, but no significant promotion effect was observed for the Δ*crp* mutant and Δ*cymA* mutant (Figure 6). These results further demonstrated that the regulation of Crp to the AY decolorization efficiency is achieved by regulating the members of electron transport chains including CymA.

### Analysis of the Electron Transport Pathway From CymA to AY

**FIGURE 7:**
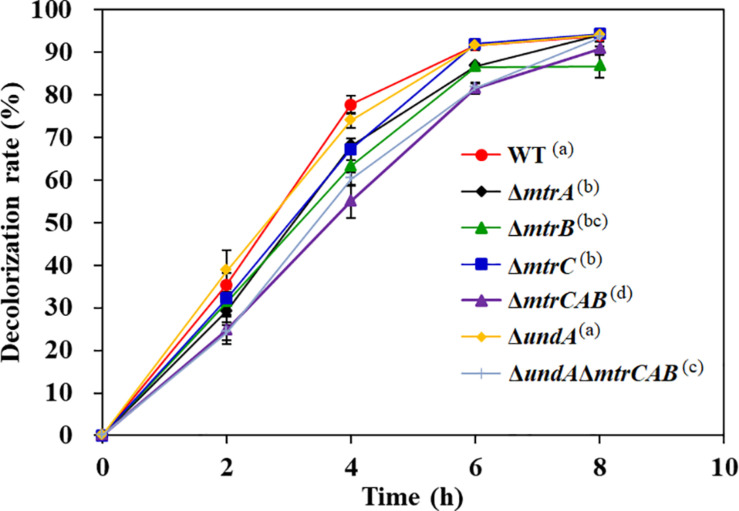
The decolorization of 200 mg/L AY by the WT and Mtr pathway mutants. Values are means ± SD (*n* = 3). The decolorization rates of different strains at 4 h were analyzed by ANOVA with the Tukey–Kramer comparison test (*p* < 0.05).

CymA is an electron transfer hub supporting multi-branched respiratory chains (Marritt et al., 2012). Recent studies showed that the Mtr respiratory pathway of *S. oneidensis* MR-1 not only participates in the dissimilatory reduction of multiple metal ions (Toporek et al., 2019) but also plays a critical role in the decolorization process of a variety of textile dyes (Cai et al., 2012). Genome analysis found that an *mtr*-like gene cluster exists in the genome of *S. putrefaciens* CN32, including MtrC, MtrA, MtrB, and UndA, and their amino acid sequences exhibit 53.5, 88.6, 83.4, and 26.6% similarity to MtrC, MtrA, MtrB, and OmcA, respectively, in *S. oneidensis* MR-1. To determine whether the Mtr respiratory pathway is involved in AY decolorization in *S. putrefaciens* CN32, the in-frame deletion mutants Δ*mtrC*, Δ*mtrA*, Δ*mtrB*, and Δ*undA* and the triple mutants Δ*mtrCAB* and Δ*undA*Δ*mtrCAB* were constructed. As shown in Figure 7, Δ*mtrC*, Δ*mtrA*, Δ*mtrB*, Δ*undA*, Δ*mtrCAB*, and Δ*undA*Δ*mtrCAB* showed 10.6, 9.8, 14.5, 3.8, 22.8, and 17.6% decreases in the decolorization efficiency of AY at 4 h, respectively, indicating that the Mtr respiratory pathway only partially contributes to the transfer of electron required for the AY decolorization process in *S. putrefaciens* CN32. This is consistent with the results previously reported (Xiao et al., 2012). At the same time, we also noticed that the blocking of the Mtr pathway could not completely inhibit the AY decolorization efficiency and that the influence of the Mtr pathway on the decolorization of MR-1 was more significant than that of CN32. In *S. putrefaciens* CN32, the decolorization efficiency of Mtr mutants (except Δ*cymA* mutant) decreased only about 20% compared with the WT strain; however, in *S. oneidensis* MR-1, a decrease of more than 60% in decolorization efficiency was reported (Xiao et al., 2012), indicating that MtrA, MtrB, and MtrC only partially contribute to the AY decolorization in *S. putrefaciens* CN32. This is similar to the latest published result, which showed that MtrA, MtrB, and MtrC did not play a major role in the decolorization of methyl orange by *S. putrefaciens* CN32 under microaerobic conditions in 96-well plates (Min et al., 2020). Thus, *S. putrefaciens* CN32 exhibits a more complex electron transfer process than did *S. oneidensis* MR-1 in the decolorization of azo dyes. Therefore, it is necessary to identify other main electron transfer pathways from the CymA electron transfer hub to azo dye acceptors in future studies.

### Mechanism of Crp Regulating the AY Decolorization in *S. putrefaciens* CN32

**FIGURE 8:**
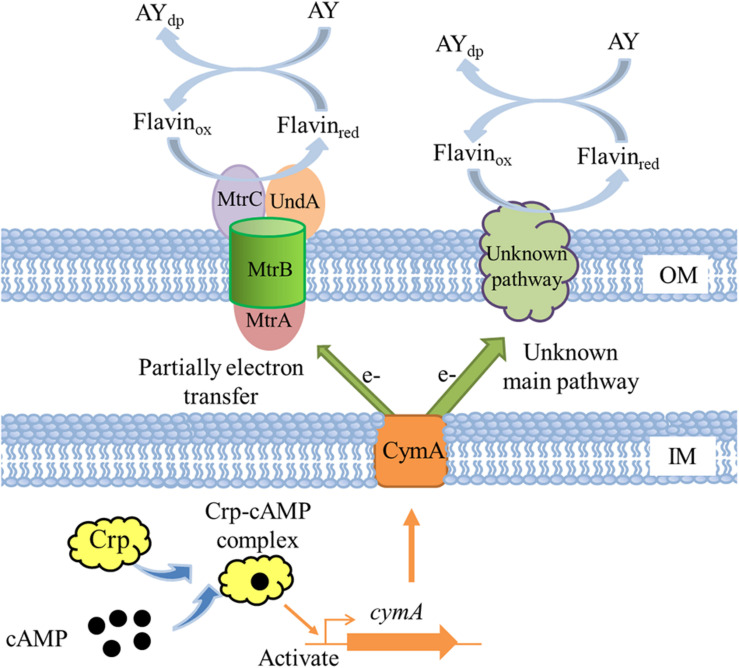
A proposed molecular model of Crp regulating AY decolorization in *S. putrefaciens* CN32. OM indicates outer membrane; IM indicates inner membrane; AY_dp_ indicates degradation products of acid yellow.

A molecular mechanism model of Crp regulating AY biodecolorization was proposed in Figure 8. Crp and cAMP form a complex and then directly activate the transcription of *cymA*; MtrA, MtrB, and MtrC partially contribute to electron transfer from cells to AY acceptors, and other major electron transfer pathways from CymA to dye molecules need to be identified in future studies; flavin acts as a shuttle to accelerate the electron transfer from cells to dye molecules through the switch between the oxidation state and reduction state. AY dye molecules are decomposed into colorless degradation products under the action of electron reduction. Based on the above molecular regulation mechanism, we tried to improve the decolorization ability of *S. putrefaciens* CN32 by overexpression of *cymA* in the WT strain (O-*cymA* mutant). The result showed that the decolorization ability of the O-*cymA* mutant was slightly improved compared to that of the WT strain. The above results indicate that Crp is necessary to activate the expression of *cymA*, thereby promoting AY decolorization through accelerating electron transfer from cells to dye molecules.

## Conclusion

In this study, AY was selected as an electron acceptor to reveal the molecular mechanism of *S. putrefaciens* CN32 decolorization of azo dyes. By constructing a transposon mutant library, the cAMP receptor protein Crp was identified as a necessary regulator for AY decolorization in *S. putrefaciens* CN32. Crp can directly bind to the promoter region of the *cymA* gene and activate the expression of the *cymA* gene, thereby supporting AY decolorization. MtrA, MtrB, and MtrC partially contribute to the electron transfer from CymA to AY molecules, and other major electron transfer pathways need to be identified in future studies. Furthermore, the overexpression of *cymA* could slightly enhance the decolorization efficiency of AY in *S. putrefaciens* CN32. This study will help us understand the molecular mechanism of azo dye decolorization in other *Shewanella* strains.

## Data Availability Statement

The raw data supporting the conclusions of this article will be made available by the authors, without undue reservation.

## Author Contributions

WL and CL designed the research. WL and YC performed decolorization experiments. YC and XZ operated gene deletion and complementation experiments. JL and JZ carried out protein expression and purification experiments. YC, SW, and DS performed RNA extraction, RT-qPCR and DNase I footprinting assays. WL, CL, and DS wrote and revised the manuscript. All authors contributed to the article and approved the submitted version.

## Conflict of Interest

The authors declare that the research was conducted in the absence of any commercial or financial relationships that could be construed as a potential conflict of interest.

## Footnotes

1 https://fruitfly.org/seq_tools/promoter.html
